# Recurrent decompression sickness and late repermeabilization of patent foramen oval closure prosthesis: a diver’s dilemma—case report

**DOI:** 10.1093/ehjcr/ytae371

**Published:** 2024-07-31

**Authors:** Antoine Deney, Olivier Lairez, Mathieu Coulange, Béatrice Riu, Jennifer Hunt

**Affiliations:** Department of Cardiology, Rangueil University Hospital, 1, Avenue Jean Poulhès, TSA 50032, 31059 Toulouse, France; Medical School, Toulouse III Paul Sabatier University, Toulouse, France; Department of Hyperbaric Medicine, Sainte Marguerite Hospital, APHM, Marseille, France; Inserm 1263—Inra 1260, Team V: Adenosinergic System and Cardiovascular Disease, Bd Jean Moulin, Marseille, France; Critical Care Unit, University Hospital of Purpan, 31300 Toulouse, France; Hyperbaric Center, University Hospital of Purpan, 31300 Toulouse, France

**Keywords:** Decompression sickness, Patent foramen oval, PFO closure, Scuba diving, Prosthesis patency, Right-to-left shunt, Case report

## Abstract

**Background:**

Decompression sickness (DCS) is a well-known risk associated with scuba diving, particularly in people with right-to-left shunt, such as patent foramen oval (PFO). Herein, we present a unique case of late PFO permeabilization after closure.

**Case summary:**

A 26-year-old male diver was diagnosed with DCS following a dive at 36 m. He underwent PFO closure with a STARFLEX® prosthesis. Ten years later, the patient was presented with recurrent manifestations suggestive of DCS. The performed diagnostic work-up detects a permeabilization of the implanted prosthesis, and he was treated with a conservative approach.

**Discussion:**

This case highlights the challenges in the management of PFO in divers and raises concerns about the long-term efficiency of PFO closure and the impact of diving-related factors on prosthesis patency.

**Learning points**

- Physician’s awareness on the long-term risk of repermeabilization after patent foramen oval PFO closure in divers.
- Lifelong regular monitoring of PFO closure is mandatory for divers.
- Dive profile and repeated dives may impact on the durability of the PFO closure.

## Introduction

**Figure 1:**
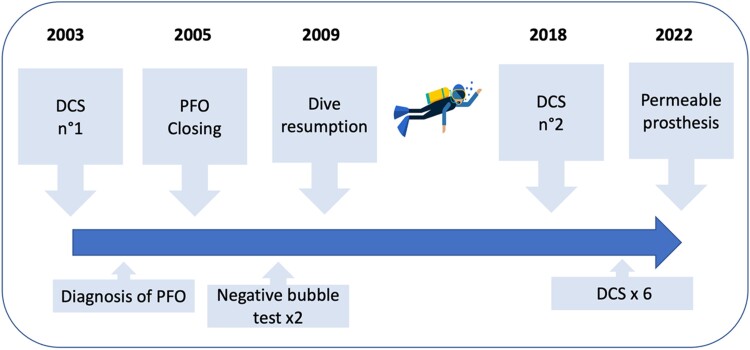
Graphical abstract resuming the timeline of the reported case.

Decompression sickness (DCS) is a recognized medical condition among scuba divers. It is characterized by a broad spectrum of symptoms thought to be caused by bubbles of air in the tissue. Patent foramen oval (PFO), a common anatomical variant in the general population, has been implicated in the pathogenesis of DCS.^1^ The closure of PFO may be recommended in patients with large shunts to mitigate the risk of DCS recurrence.^2^ Herein, we report a case of recurrent DCS symptoms in a proficient scuba diver following a successful PFO closure with a prosthesis (*Figure 1*).

## Case presentation

A 26-year-old man suffered inner ear DCS with rotatory vertigo and vomiting after diving to a maximum depth of 36 m for a total duration of 28 min in Ajaccio, France, in August 2003. The patient is previously healthy with no significant past medical history or reported cardiovascular risk factors. The patient underwent an otolaryngological assessment, which revealed no abnormalities. A brain magnetic resonance imaging was also unremarkable. Effective treatment was achieved through normobaric and hyperbaric oxygen therapies along with antiplatelet therapy using 75 mg of lysine acetylsalicylate.

**Figure 2:**
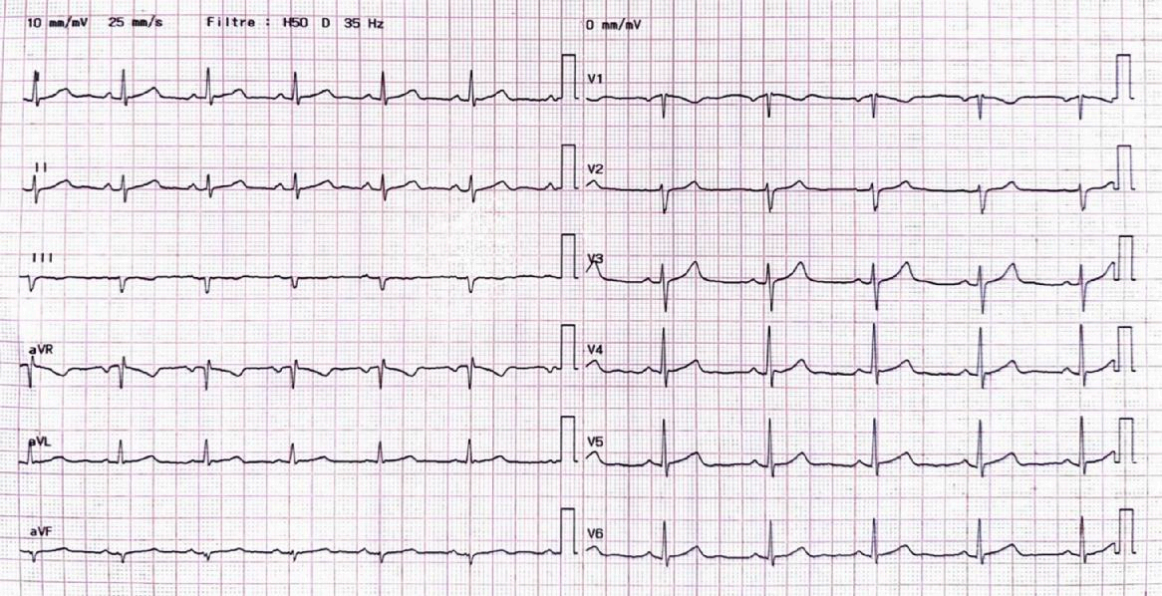
Electrocardiogram showing no conduction and repolarization abnormalities.

**Figure 3:**
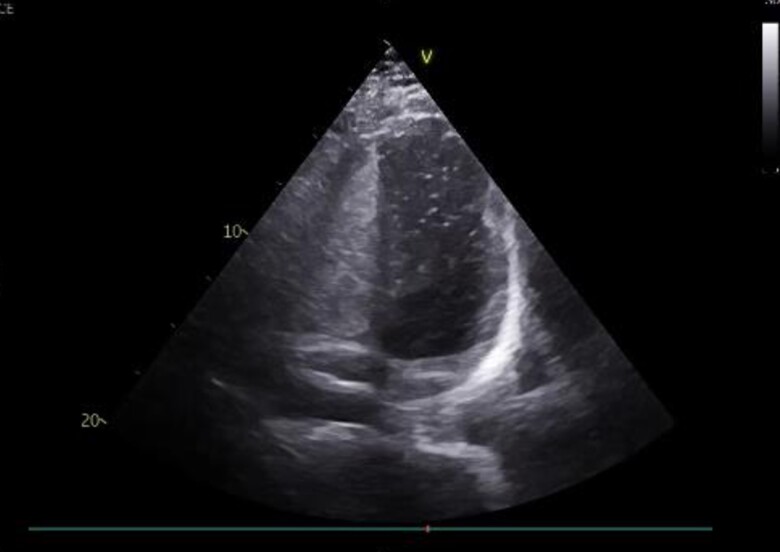
A bubble study transthoracic echocardiogram showing right-to-left shunt.

**Figure 4:**
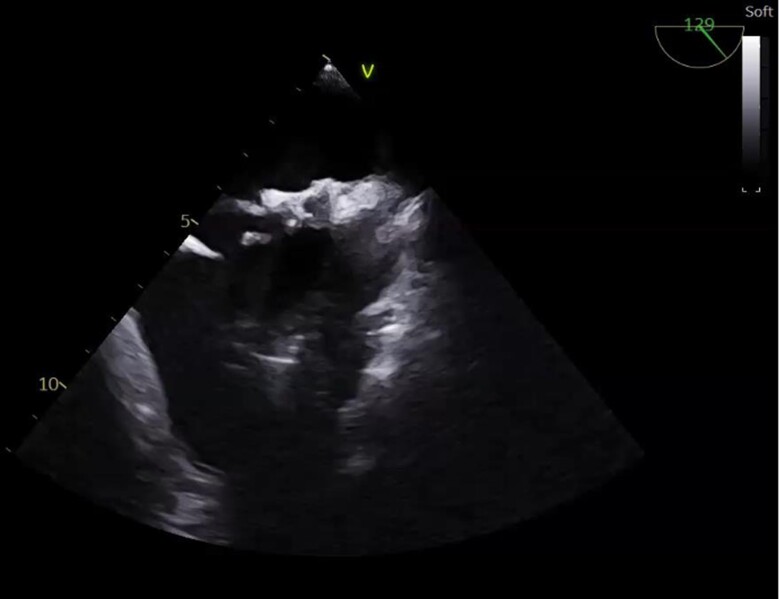
Transthoracic echocardiogram showing the *STARFLEX® prosthesis* with the residual shunt.

Upon returning to his residence in Toulouse, France, the patient sought a consultation with a cardiologist. The physical examination was unremarkable. The use of performance-enhancing drugs was excluded based on detailed history taking and physical examination for the detection of signs indicative of concealed use of performance-enhancing drugs.^3^ The electrocardiogram showed regular sinus rhythm at a rate of 59 b.p.m., an incomplete right bundle branch block, and no other conduction abnormalities or repolarization disorders (*Figure 2*). As part of the evaluation of the decompression accident, transthoracic echocardiography (TTE) (*Figure 3*) and transoesophageal echocardiogram (TEE) bubble studies (*Figure 4*) were performed, revealing an aneurysmal interatrial septum and a PFO with spontaneous positive contrast enhancement, without the need for a Valsalva manoeuver. No intracardiac thrombus was observed, and the cardiac valves appeared normal. The ascending aorta did not show signs of dilation.

After multidisciplinary discussions and considering the patient’s desire to continue diving, a decision was made to proceed with PFO closure. Two years later, the patient underwent implantation of a 33 mm STARFLEX® prosthesis in the interatrial septum. At the 7-month follow-up, a repeat TEE demonstrated excellent placement of the prosthesis, providing near-complete coverage of the interatrial aneurysm. No residual shunt was detected on colour Doppler examination. Contrast testing revealed no spontaneous bubble passage, even with repeated Valsalva manoeuvers. The maximum diameter of the ascending aorta remained within normal limits, measuring 31 mm. Based on the favourable findings, antiplatelet therapy was discontinued, and the patient was cleared to resume unrestricted diving activities. From 2009 onwards, the patient resumed regular diving without encountering any difficulties. A 2-year follow-up was performed with another TTE showing the absence of a residual shunt.

In 2018, while diving in Bali, Indonesia, the patient experienced pruritus of the abdomen and diffuse violaceous mottling over his trunk after his 2nd dive of the day, reaching a maximum depth of 30 m, and adhering to safety stops. The symptoms improved after aspirin administration, subsiding within 2 h. Since then, the patient has had six similar episodes following dives of varying depths, including dives reaching up to 60 m with nitrox.

He has never sought medical advice for these episodes. After the 7th event, he consulted his cardiologist, who performed a TEE bubble study with Valsalva manoeuver, revealing a spontaneously provoked shunt Grade 3 indicating permeabilization of the PFO. The decision was made to refrain from recommending a secondary PFO closure and to enforce diving restrictions in alignment with the French Underwater Federation.

## Discussion

To our knowledge, this is the 1st reported case of prosthesis permeabilization occurring more than 15 years after implantation on a large PFO, accompanied by recurrent DCS in a diver who continued unrestricted diving for an extended period. The prevalence of a PFO in the general population is 25–30%,^4^ with a particular feature being an increase in patency over time in divers.^5^ The link between the presence of PFO and the onset of a DCS is now widely accepted.^6^ Large PFOs, particularly those with a large shunt, present an increased risk of DCS.^7^ Consequently, closure of these PFOs may be recommended, particularly for large Grade 3–4 PFOs, to minimize the risk of recurrence.^2^

The presence of cutaneous manifestations, such as livedo reticularis or cutis marmorata, following dives should be considered as a significant DCS event associated with arterial occlusion or embolism.^8^ A recent retrospective study demonstrated a correlation between these cutaneous manifestations and right-to-left shunting, with 100% of divers presenting cutis marmorata having a right-to-left shunt, 83% of which were associated with an intracardiac shunt.^9^ This phenomenon is believed to result from emboli in the brainstem, affecting the autonomic nervous system’s regulatory site and Vaso motility regulation in cutaneous vessels, similar to neurological diseases associated with livedo reticularis.^8–10^ Therefore, evaluating the presence of a shunt is appropriate in this clinical case.

The favourable outcome of the prosthesis at the 7-month and 2-year follow-up, respectively, with a well-sealed device and no evidence of residual shunt, raises questions about the potential causes of the observed permeabilization. One possibility is a false-negative result on the initial TEE bubble study, indicating residual shunting despite the absence of abnormal findings. However, the performance of TEE under local anaesthesia allowing an optimal patient cooperation^11^ and the fact that the patient has never presented DCS until 2018 make this hypothesis less likely.

Another consideration is whether the STARFLEX® prosthesis itself could be responsible for a sealing failure and increased risk of decompression sickness. However, existing literature does not report cases of recurrent gas embolism related to diving in the context of this prosthesis. While an increased risk of atrial fibrillation or thrombus formation has been observed in patients receiving the STARFLEX® prosthesis for cryptogenic stroke,^12^ there is limited evidence suggesting a performance defect compared with the Amplatzer prosthesis.^13^ Although the presence of a residual shunt cannot be completely ruled out, the extremely low rate of events and the absence of visual defects on TEE make this hypothesis less likely. Notably, the patient exhibited significant dilation of the ascending aorta, reaching 41 mm after 15 years. The association between PFO and ascending aortic dilation is well established.^14^ Although it is not proven that diving itself increases aortic size, the repeated pressure variations during diving may contribute to aortic dilation and alteration of the interatrial septum’s geometry, potentially leading to PFO reopening. Additionally, prolonged right-sided pressures and the performance of Valsalva manoeuvers during diving may exacerbate the presence and progression of PFO in divers.^5^

Lastly, we emphasize that TTE is the first diagnostic step for detecting PFO. Existing literature reported the diagnostic value of TTE for PFO. The reported sensitivity and specificity of TTE in detection of PFO were high up to 88% and 97%, respectively.^15^ However, the accuracy of TTE depends on the experience of the sonographer. An experienced sonographer may be able to discover more clues associated with PFO. We also mention the limitations of TTE in patients with a small right-to-left shunt. Transoesophageal echocardiogram with the addition of agitated saline contrast remains the gold standard imaging modality for the identification of PFO.

## Conclusion

In conclusion, this case highlights the dilemma of PFO closure in divers. While closure initially appeared beneficial, the occurrence of permeabilization and recurrent DCS raises questions about the long-term efficiency of the procedure. The influence of diving-related factors on prosthesis patency and the need for ongoing monitoring and evaluation in divers with PFO warrant further investigation. Improved understanding of the interaction between PFO closure, diving physiology, and prevention of DCS recurrence in high-risk individuals is crucial for informed decision-making and optimal management strategies.

## Lead author biography


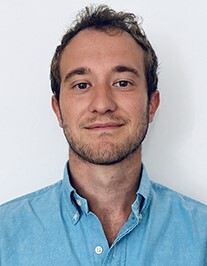


Antoine Deney is a cardiologist and currently an assistant chief resident in advanced heart failure at Toulouse Rangueil University Hospital. His career centres on sports cardiology, hyperbaric medicine, advanced heart failure, and multimodal cardiovascular imaging. He has contributed to multiple research projects, 20 of which are currently listed on PubMed.

##  

**Consent:** The authors confirm that written consent for submission and publication of this case report including images and associated text has been obtained from the patient in line with COPE guidelines.

**Funding:** Centre Hospitalier Universitaire de Toulousel provides the article processing fees.

## Data Availability

The data underlying this article are available in the article.
